# Evaluating biventricular diastolic function using cardiovascular magnetic resonance 4d-flow derived E/e’

**DOI:** 10.1093/ehjimp/qyag039

**Published:** 2026-03-09

**Authors:** Leonard Grob, Jacopo Soldini, Stephanie Keser, Davide Colatruglio, Louis Setz, Anna C Zimmermann, Dario Kaiser, Bernd Jung, Adrian T Huber, Hendrik von Tengg-Kobligk, Martina Boscolo Berto, Matthias Wilhelm, Christoph Gräni, Dominik P Guensch, Kady Fischer

**Affiliations:** Department of Anaesthesiology and Pain Medicine, Inselspital, Bern University Hospital, University of Bern, Freiburgstrasse 10, Bern 3010, Switzerland; Department of Anaesthesiology and Pain Medicine, Inselspital, Bern University Hospital, University of Bern, Freiburgstrasse 10, Bern 3010, Switzerland; Department of Anaesthesiology and Pain Medicine, Inselspital, Bern University Hospital, University of Bern, Freiburgstrasse 10, Bern 3010, Switzerland; Department of Anaesthesiology and Pain Medicine, Inselspital, Bern University Hospital, University of Bern, Freiburgstrasse 10, Bern 3010, Switzerland; Department of Anaesthesiology and Pain Medicine, Inselspital, Bern University Hospital, University of Bern, Freiburgstrasse 10, Bern 3010, Switzerland; Department of Anaesthesiology and Pain Medicine, Inselspital, Bern University Hospital, University of Bern, Freiburgstrasse 10, Bern 3010, Switzerland; Department of Anaesthesiology and Pain Medicine, Inselspital, Bern University Hospital, University of Bern, Freiburgstrasse 10, Bern 3010, Switzerland; Department of Diagnostic, Interventional and Paediatric Radiology, Inselspital, Bern University Hospital, University of Bern, Bern, Switzerland; Translation Imaging Center (TIC), Swiss Institute for Translational and Entrepreneurial Medicine, Bern, Switzerland; Department of Diagnostic, Interventional and Paediatric Radiology, Inselspital, Bern University Hospital, University of Bern, Bern, Switzerland; Department of Radiology and Nuclear Medicine, Lucerne Cantonal Hospital, University of Lucerne, Lucerne, Switzerland; Department of Diagnostic, Interventional and Paediatric Radiology, Inselspital, Bern University Hospital, University of Bern, Bern, Switzerland; Translation Imaging Center (TIC), Swiss Institute for Translational and Entrepreneurial Medicine, Bern, Switzerland; Department of Cardiology, Inselspital, Bern University Hospital, University of Bern, Bern, Switzerland; Department of Cardiology, Inselspital, Bern University Hospital, University of Bern, Bern, Switzerland; Department of Cardiology, Inselspital, Bern University Hospital, University of Bern, Bern, Switzerland; Department of Anaesthesiology and Pain Medicine, Inselspital, Bern University Hospital, University of Bern, Freiburgstrasse 10, Bern 3010, Switzerland; Department of Diagnostic, Interventional and Paediatric Radiology, Inselspital, Bern University Hospital, University of Bern, Bern, Switzerland; Department of Anaesthesiology and Pain Medicine, Inselspital, Bern University Hospital, University of Bern, Freiburgstrasse 10, Bern 3010, Switzerland

**Keywords:** cardiovascular magnetic resonance, 4D flow, diastolic dysfunction, myocardial strain, e/e’, right ventricle

## Abstract

**Aims:**

Cardiovascular magnetic resonance (CMR) imaging is a key modality for characterizing heart diseases, but is limited in assessing diastolic dysfunction (DD). 4D flow CMR now enables transvalvular blood flow quantification, while biventricular tissue relaxation can be quantified through annular tissue velocity and strain on standard cine images. This study investigated the utility of 4D-CMR-derived E/e′ in evaluating biventricular diastolic function. Secondary aims included comparison with echocardiography to establish 4D-E/e′ cutoffs for detecting unknown DD.

**Methods and results:**

Diastolic transvalvular flow (4D-E) was quantified from 4D flow in 75 controls and 57 patients with cardiovascular disease. Tissue velocity (e′) was assessed using cine-derived mitral/tricuspid annular velocity, longitudinal strain rate (e′_FT-SR_), and strain velocity (e′_FT-vel_). Biventricular 4D-E/e′ was feasible across all e′ methods, and significantly higher in patients than controls (*P* < 0.05). The patients were split into two subgroups: one with echocardiographic graded DD to derive CMR cutoffs, and a second with unassessed diastolic function. 4D-E/e′ using annular velocity best distinguished patients with echocardiography-confirmed DD in the left (AUC = 0.90 ± 0.05, *P* < 0.01) and right heart (AUC = 0.81 ± 0.07, *P* < 0.01). Among patients without a diastolic assessment, 71% were identified with abnormal left ventricular diastolic function and 61% with abnormal right ventricular diastolic function when stratified against the lower 4D-E/e’ cutoffs.

**Conclusion:**

4D-E/e′, integrating transvalvular flow and tissue velocity, is feasible for biventricular diastolic function assessment. CMR identified previously unrecognized biventricular diastolic abnormalities in patients with cardiovascular disease, suggesting 4D-E/e′ may be a valuable tool for early detection and referral for further diastolic testing.

## Graphical Abstract

**Graphical Abstract:**
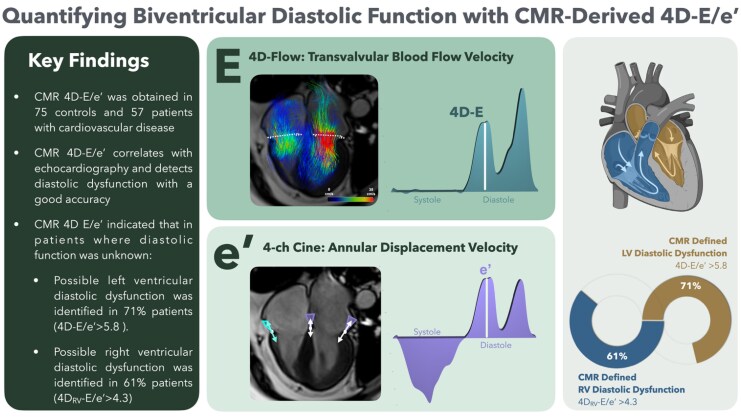
CMR = cardiovascular magnetic resonance, ch = chamber, LV = Left ventricle, RV = right ventricle.

## Introduction

Cardiovascular magnetic resonance (CMR) imaging has become an invaluable tool for the evaluation of cardiovascular diseases (CVD), with a rise in referral rates outpacing those for invasive stress testing.^1^ While assessments of tissue characterization and systolic function remain core measures in the clinical reports, CMR is limited in its ability to quantify diastolic function. Detecting diastolic dysfunction (DD) during a cardiac workup is important, as it is often one of the first signs of impaired left ventricular (LV) function, appearing before a decline in systolic ejection fraction occurs.^2^ DD is an independent predictor for poor cardiovascular outcomes for many subtypes of CVD, beyond measurements of myocardial ischaemia and ejection fraction,^3–5^ and is established as a crucial precursor in the development of heart failure if left untreated. Although CMR is not the tool of choice to assess DD, its versatility makes it ideal for detecting signs of DD secondary to other CVD when patients are already undergoing a CMR exam.

One of the most clinically applied imaging parameters for DD assessment is the E/e’ ratio, a measurement common in echocardiography exams yet not in CMR. This imaging parameter particularly relies on both measurements of blood flow velocity through the atrioventricular valves during early (E) and late (A) diastole, as well as the corresponding tissue velocity of the valvular annulus (e’ and a’) to quantify impaired relaxation.^6^ For the latter factor, multiple CMR techniques have been shown to assess diastolic relaxation of tissue, either by measuring the annular velocity or by measuring relaxation of the ventricular wall.^7–10^ Another CMR approach involves quantifying mitral and tricuspid annular excursion velocities on a single four chamber cine image. This tool has already been validated against M-mode echocardiography^11^ and allows for distinguishing patients with different DD grades.^9,10^ Yet it is the assessment of the transvalvular blood flow (E and A), that currently represents a significant limitation in CMR. Quantification of transvalvular blood flow with CMR is traditionally performed with two-dimensional (2D) phase-contrast imaging and is comparable to echocardiography.^12,13^ However, there are limitations with 2D flow when quantifying the mitral and tricuspid valves, as due to their significant motion a perpendicular plane to the orifice throughout the entire cardiac cycle is not often feasible. With the introduction of whole heart 4D-CMR imaging (4D flow), the valves throughout the entire cardiac cycle can be tracked accounting for their through-plane and angular motion.^14,15^

Within the last five years, the potential of left heart 4D transmitral flow (4D-E and 4D-A) for discriminating left ventricular DD has been introduced,^16–18^ demonstrating this is a burgeoning field. Yet, few studies have derived a 4D-E/e’ ratio, that combines both 4D transvalvular blood flow and tissue velocities.^15,19,20^ These exciting new publications have introduced 4D-E/e’ as a novel marker. However, the rare studies assessing DD relied only on static planes, which do not account for through-plane motion or fully leverage the benefits of the 4D dataset (differences in techniques described in Supplementary data online, *Table S1*). Therefore, further progress may be achieved by using valve-tracking techniques for transvalvular flow, and by determining if the 4D-E/e’ ratio can be improved using different CMR-based tissue relaxation parameters, as described above, for the e’ component of the ratio. Moreover, the feasibility of right ventricular 4D-E/e’ has not yet been evaluated, despite the fact that a key advantage of whole heart 4D flow imaging is that right heart function can be simultaneously assessed.

Thus, the aim of this study was to assess the utility of deriving CMR-based 4D-E/e,’ combining 4D flow with tissue relaxation and strain rate measurements of both the left and right ventricle, to evaluate biventricular diastolic function in healthy controls and patients with CVD. Secondary aims were to compare these measurements to echocardiography, a pragmatic clinical tool for assessing diastolic function, and to generate cutoff values for 4D-E/e’ to assess the prevalence of unknown DD in the CVD patients.

## Methods

**Figure 1:**
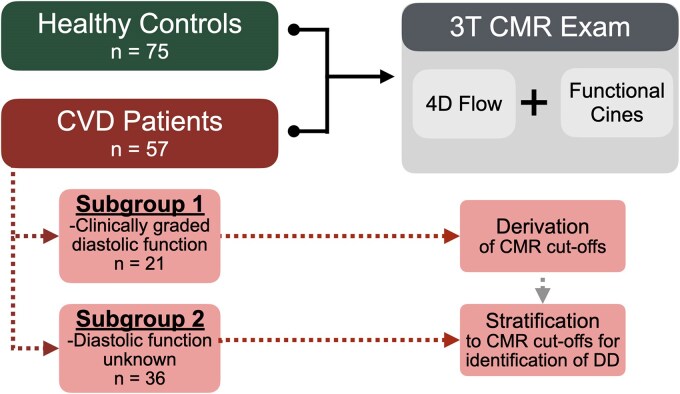
Study design. A total of 132 participants, 75 healthy controls and 57 patients with cardiovascular disease (CVD), underwent a 3 tesla (T) CMR exam. The CVD patient group was divided into two subgroups. Subgroup 1 consisted of 21 patients with a clinical assessment of diastolic dysfunction, which was used to validate the detection of diastolic dysfunction against echocardiography and to derive CMR cutoff values. Subgroup 2 were the CVD patients with an unknown diastolic status, and patients in this subgroup were stratified against the cutoffs to quantify the prevalence of unknown diastolic dysfunction based on CMR.

The study population included 75 healthy controls with no cardiovascular or respiratory disease, and 57 CVD patients who underwent a research-indicated CMR scan with 4D flow. CVD patients had a confirmed diagnosis of heart failure and/or chronic coronary syndromes and had attended an appointment or undergone an intervention/assessment with cardiology or cardiovascular anaesthesiology services. CVD patients were further divided into two subgroups: those with a clinical assessment of diastolic function, and those with undefined diastolic function (*Figure 1*). Written consent for secondary use of data was obtained from all participants. The study protocol was reviewed and approved by the ethics committee of the canton of Bern (MACDAVD, #2020_01258).

### CMR imaging protocol

Imaging was performed on a 3.0-Tesla clinical scanner (MAGNETOM Prisma or Skyra, Siemens Healthineers, Germany). Balanced steady-state free precession cine images were acquired in long-axis and short-axis views aligned to the LV, with a temporal resolution of 25–30 phases per cardiac cycle. A 4D flow dataset covering the thoracic cavity was obtained with a diaphragmatic respiratory navigator (sequence parameters are detailed in the Supplementary data online, methods).^21^

### CMR image analysis

**Figure 2:**
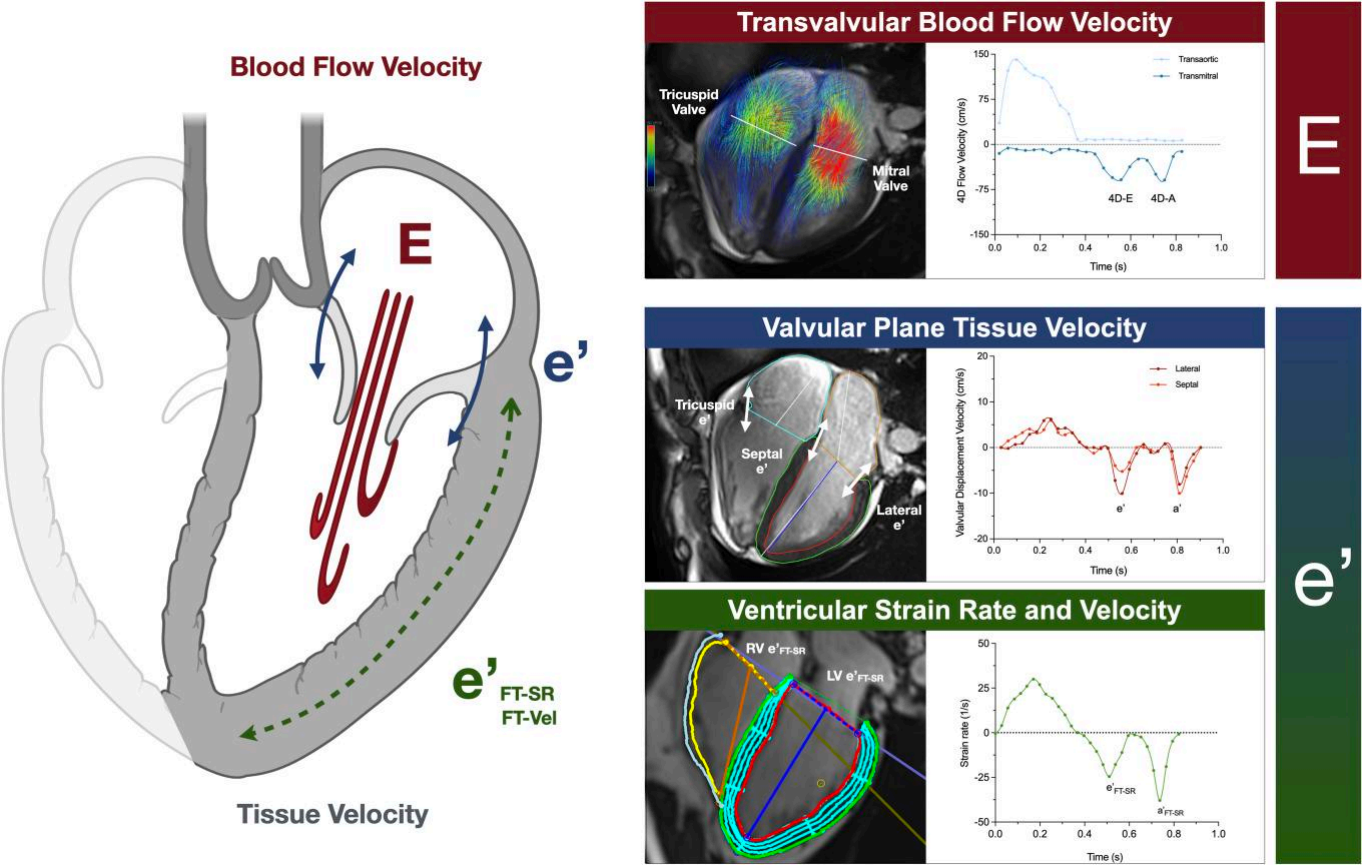
Quantifying diastolic parameters with CMR. D flow datasets provide mitral and tricuspid blood inflow velocity for early (E) and late (A) diastole (top right). Tissue velocity can be quantified from the velocity of the diastolic movement of the mitral and tricuspid planes (e’, a’, *top right*), or by relaxation of the entire ventricular wall quantified by strain rates (e’_FT-SR_, a’_FT-SR_) and velocities (e’_FT-Vel_, a’_FT-Vel_) from feature tracking strain analysis (*middle and bottom right)* on standard cines.

Image analyses were performed with Circle Cardiovascular Imaging (version 6.0, Calgary, AB, Canada). Standard volumetric parameters were calculated, with left atrial volume and left ventricular mass used to further calculate sex-adjusted CMR-derived pulmonary capillary wedge pressure (PCWP)^22^ (see Supplementary data online, *Data S3*). For transvalvular blood flow measurements 4D flow datasets were evaluated using a dynamic valve tracking tool within the 4D flow analysis module. First, anti-aliasing and threshold correction were performed, and a whole-heart mask was segmented. Using the dynamic valve tracking tool, the semilunar (aortic and pulmonary) and atrioventricular valves (mitral and tricuspid) were tracked throughout the cardiac cycle at the level of the annulus. This was performed by first using the automatic valve placement tool, which locates the valve annulus on the 4D segment based on the location of the valve on the left and right ventricular inflow and outflow tract cines. If required, the location was then manually corrected using double oblique views on the 4D segment to verify the position and angle. The valve orifice was contoured for each phase to derive peak velocities and flow rates during systole, early (4D-E) diastole, and late (4D-A) diastole (*Figure 2*).^21^ From the diastolic flow measurements, a 4D-E/A ratio was calculated for both the left and right heart. Detailed step-by-step image analysis process can be seen in Supplementary data online, *Data S2*.

For the analysis of tissue relaxation, two techniques were applied to the long-axis cines. First, a semi-automatic long-axis shortening analysis technique (Bi-Triplanar module, Circle Cardiovascular Imaging) was used to assess annular plane displacement of the tricuspid and the mitral valve on the 4-chamber cine. The lateral and septal mitral annuli and the lateral tricuspid annulus were automatically tracked throughout the entire cardiac cycle and adjusted manually if needed. For the mitral valve, lateral and septal annular displacements were averaged.^10^ Early (e’) diastolic tissue velocities were computed as the time derivative of annular excursion, with e’ being defined as the negative peak during early diastole, closely resembling the tissue Doppler-index approach in echocardiography. The second technique assessed deformation of the entire ventricular wall using feature-tracking software. Three long-axis cines (2-, 3-, and 4-chamber) were analysed for early (e’_FT-SR_) longitudinal diastolic strain rates, as well as the measurement for early (e’_FT-Vel_) longitudinal diastolic strain velocity.^23^ Three combined ratios (4D-E/e’, 4D-E/e’_FT-SR,_ and 4D-E/e’_FT-Vel_) were calculated for both ventricles, integrating blood flow velocity and the three myocardial tissue motion metrics (Video S1).

### Statistical analysis

Measures are reported as mean ± standard deviation. For the primary aim, biventricular CMR-derived diastolic values were compared between controls and CVD patients using an ANOVA, adjusting for age. For secondary analysis, LV CMR diastolic parameters were compared with analogous echocardiographic parameters in patients who had undergone echocardiography within 90 days before the CMR scan, using Pearson’s correlation. The third analysis assessed the diagnostic performance of the different CMR-derived parameters for detecting DD and provided a lower and upper cutoff. For the LV, this was done by comparing the subgroup of CVD patients with clinically graded DD to control values using a logistic regression. The area under the curve (AUC) was calculated, and diagnostic cutoffs were defined based on 80% specificity to identify a lower limit at which LV-DD is likely. As a clinical diagnosis of RV-DD is rare, diagnostic performance analysis was not feasible; therefore, a lower threshold for RV-DD was defined using the 80th percentile of values from the healthy cohort. For both ventricles, upper thresholds, indicating a higher likelihood of DD, were derived from the 97.5th percentile of the healthy cohort, consistent with conventional reference interval methods. In the final step, these cutoffs were applied to the remaining CVD patients (subgroup 2) to estimate the prevalence of undetected biventricular DD. Twelve patients were re-coded and read by a second blinded reader, and interobserver reliability was assessed using an intraclass correlation coefficient (ICC) based on a two-way mixed-effects model for absolute agreement. Statistical analysis assessing CMR-derived PCWP is detailed in the Supplementary data online, *Data S3*. A two-sided *P*-value of <0.05 was used to define statistical significance. Statistical analysis was conducted using GraphPad Prism version 10.2.2 (GraphPad Software, La Jolla, California, USA) and IBM SPSS Statistics 26 (IBM, Armonk, NY, USA).

## Results

Detailed participants characteristics are provided in Supplementary data online, *Table S2*. Out of 132 included 4D flow datasets, left heart measurements were successfully quantified in 130 cases, and right heart measurements in 132 cases. Two left heart datasets were excluded due to artefacts from artificial mitral valves. After adjusting for age, controls had a higher LV ejection fraction (59 ± 5% vs. 55 ± 11%, *P* < 0.01) and cardiac index (3.2 ± 0.8 L/min/m^2^ vs. 2.7 ± 0.8 L/min/m^2^, *P* < 0.01). Most CVD patients (89%) had a preserved LV ejection fraction ≥45% (see Supplementary data online, *Table S3*).

### Comparison of LV CMR diastolic measures between controls and CVD-patients

Transmitral blood flow velocities showed that controls had higher 4D-E (78 ± 14 cm/s vs. 65 ± 18 cm/s, *P* < 0.01) but lower 4D-A velocities (40 ± 9 cm/s vs. 58 ± 18 cm/s, *P* < 0.01) than CVD patients. No significant difference was found for the 4D-E/A ratio between the two groups (1.8 ± 0.5 vs. 1.5 ± 1.4, *P* = 0.09). Early diastolic LV relaxation was greater in healthy controls reflected by higher values for the mitral annular plane velocity (e’: 16.5 ± 4.2 cm/s vs. 8.2 ± 2.4 cm/s, *P* < 0.01), strain rate (e’_FT-SR_: 1.09 ± 0.29/s vs. 0.69 ± 0.28, *P* < 0.01) and strain velocity (e’_FT-Vel_: 38 ± 14 cm/s vs. 23 ± 12 cm/s *P* < 0.01). Consequently, all three 4D E/e’ ratios were significantly lower in controls than patients (4D-E/e’: 4.9 ± 1.2 vs. 8.6 ± 3.5, *P* < 0.01, 4D-E/e’_FT-SR_: 75 ± 22 cm vs. 113 ± 75 cm, *P* < 0.01, E/e’_FT-Vel_: 2.3 ± 0.9 vs. 4.0 ± 5.5, *P* = 0.02). *Table 1* displays the CMR diastolic measurements.

**Table 1:** CMR diastolic measurements

	Left heart			Right heart		
	Controls	CVD Patients	*P*	Controls	CVD Patients	*P*
Transvalvular blood flow velocity						
4D-E (cm/s)	78 ± 14	65 ± 18	<0.01	53 ± 9	46 ± 10	<0.01
4D-A (cm/s)	40 ± 9	58 ± 18	<0.01	32 ± 9	45 ± 17	<0.01
4D-E/A	1.8 ± 0.5	1.5 ± 1.4	0.09	1.8 ± 0.6	1.1 ± 0.6	<0.01
Tissue velocity						
e’ (cm/s)	16.5 + 4.2	8.2 ± 2.4	<0.01	16.9 ± 5.6	9.4 ± 4.3	<0.01
e’_FT-SR_ (/s)	1.1 ± 0.3	0.7 ± 0.3	<0.01	1.3 ± 0.4	1.0 ± 0.4	<0.01
e’_FT-vel_ (cm/s)	38 + 14	23 ± 12	<0.01	51 ± 20	41 ± 21	<0.01
Transvalvular blood flow/tissue velocity ratios						
4D-E/e’	4.9 ± 1.2	8.6 ± 3.5	<0.01	19 ± 6	29 ± 11	<0.01
4D-E/e’_FT-SR_ (cm)	75 ± 22	113 ± 75	<0.01	255 ± 122	271 ± 123	0.02
4D-E/e’_FT-vel_	2.3 ± 0.9	4.0 ± 5.5	0.02	7.1 ± 3.9	7.0 ± 3.6	0.02

Mean ± SD of healthy controls and patients. All comparisons are adjusted for age.

e’, average early diastolic mitral/tricuspid annular tissue velocity; e’_FT-SR_, early diastolic strain rate; e’_FT-vel_, early diastolic strain rate velocity (reported as the |absolute| value); CVD, cardiovascular disease.

### Comparison to echocardiography

**Figure 3:**
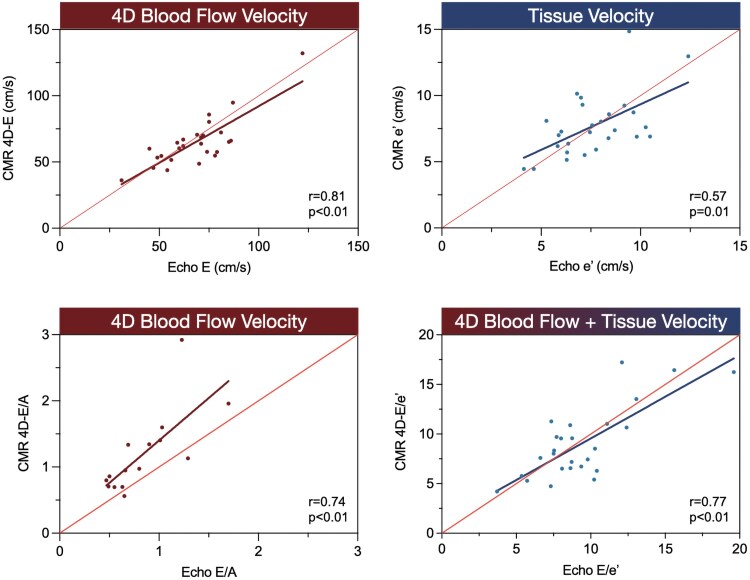
CMR mitral inflow and tissue velocity measurements correlate to echocardiography. All figures depict the correlation from the cardiovascular magnetic resonance (CMR) measurements (y-axis) to the corresponding echocardiography (echo) findings (x-axis).

Twenty-eight patients had recent echocardiography results (<90 days), where at least one diastolic measurement was provided (with or without a clinical diagnosis). Mitral inflow (E and A) and tissue Doppler mitral measurements (e’, E/e’) could be compared with the CMR values. Strong correlations between CMR and corresponding echocardiography measures were found for E (r = 0.81, *P* < 0.01) and E/e’ (r = 0.77, *P* < 0.01) with moderate correlations for e’ (r = 0.57, *P* < 0.01) and E/A (r = 0.74, *P* < 0.01) as can be seen in *Figure 3*.

### 4D flow for discriminating LV-DD

**Figure 4:**
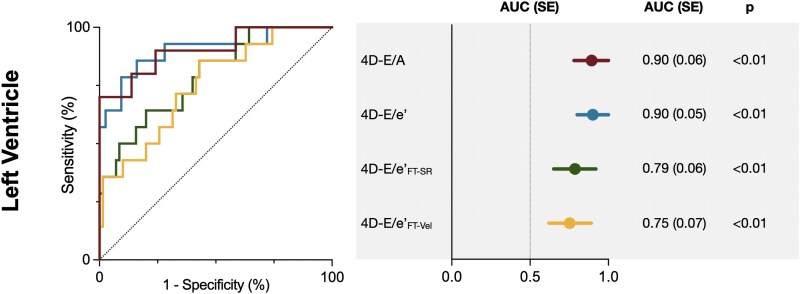
4D CMR discriminates patients with diagnosed diastolic dysfunction. Receiver operating curves (left) plotted alongside the area under the curve (AUC, right) demonstrate the ability of the CMR 4D flow ratios to discriminate patients with clinically diagnosed diastolic dysfunction (≥grade I) in comparison to control values. CMR = cardiovascular magnetic resonance.

For the discrimination of DD, only patients with a clinical diagnosis of DD were included in the analysis. Of the 28 patients with echocardiography results, diastolic function was not graded by the cardiologist in seven patients, while the remaining 21 received a clinical grade: 6 were classified as having normal diastolic function, and 15 were given a clinical diagnosis of DD of at least grade I or higher (see Supplementary data online, *Table S2*). Using this clinical diagnosis as reference, CMR-derived tissue measurements (see Supplementary data online, *Table S4*), transmitral flow velocities as well as the left heart ratios effectively discriminated patients with DD from controls (*Figure 4*). The strongest diagnostic performance was observed for 4D-E/e’ (AUC = 0.90 ± 0.05, *P* < 0.01) and 4D-E/A (AUC = 0.90 ± 0.06, *P* < 0.01). The two ratios derived from feature tracking also performed well, though with lower AUC’s (E/e’_FT-SR_: AUC = 0.79 ± 0.06, *P* < 0.01, E/e’_FT-Vel_: AUC = 0.75 ± 0.07, *P* < 0.01). Cutoff values for each parameter are displayed in *Table 2*. Specifically, for 4D-E/e’, a lower threshold of 4D-E/e’>5.8 derived from the AUC curves yielded 80% specificity and 86% sensitivity. An upper limit of 4D-E/e’>7.3 was derived from the 97.5th percentile reference intervals (97% specificity, 57% sensitivity). The second subgroup of CVD patients (*n* = 36), those without a clinical assessment of DD, were then stratified against these cutoffs. Based on this lower cutoff, 71% of ungraded CVD patients were found to have abnormal LV diastolic function (4D-E/e’>5.8), with 51% exceeding the upper cutoff (4D-E/e’>7.3). Similar assessments using only healthy controls older than 50 years as reference can be observed in Supplementary data online, *Table S5*.

**Table 2:** CMR derived cut offs

	Lower cutoff			Upper cutoff		
	Cutoff	Specificity (%)/ Sensitivity (%)	CMR defined DD (*n*, %)	Cutoff	Specificity (%)/ Sensitivity (%)	CMR defined DD (*n*, %)
Left ventricle						
4D-E/e’	>5.8	80/86	25/35 (71%)	>7.3	97/57	18/35 (51%)
4D-E/e’_FT-SR_ (cm)	>90	80/64	17/34 (50%)	>123	99/36	17/34 (21%)
4D-E/e’_FT-vel_	>2.9	80/50	15/32 (47%)	>4.8	99/29	5/32 (16%)
4D-E/A	<1.4	79/80	11/17 (65%)	<1.2	97/70	7/17 (41%)
Right ventricle						
4D_RV_-E/e’	>4.3	—	22/36 (62%)	>5.9	—	18/36 (51%)
4D_RV_-E/e’_FT-SR_ (cm)*	>52	—	15/33 (43%)	>103	—	1/33 (3%)
4D_RV_-E/e’_FT-vel*_	>1.6	—	9/28 (26%)	>3.3	—	1/28 (3%)
4D_RV_-E/A	<1.3	—	9/22 (26%)	<0.9	—	4/22 (11%)

e’, average early diastolic mitral/tricuspid annular tissue velocity; e’_FT-SR_, early diastolic strain rate; e’_FT-vel_, early diastolic strain rate velocity.

Lower cutoffs are generated based on 80% sensitivity for the left heart, or 80th percentile of healthy control values for the right heart. Upper cutoffs are based on the 97.5th percentile of healthy controls for both ventricles.

### Sex-adjusted calculations of CMR-derived PCWP

Additional analyses, detailed in Supplementary data online, Data  *S3*, demonstrate that sex-adjusted CMR-PCWP was significantly lower in controls than patients (17.0 ± 2.2 mmHg vs. 18.4 ± 4.2 mmHg, *P* < 0.01). Participants with elevated CMR-PCWP had a higher 4D-E/e’ (7.3 ± 3.7) than patients with CMR-PCWP < 17.5 mmHg (5.9 ± 2.4, *P* = 0.02), and 4D-E/e’ demonstrated a non-significant association for discriminating patients with elevated CMR-PCWP (AUC = 0.60, *P* = 0.054).

### RV measurements

Standard RV function assessments showed that 91% of CVD patients had preserved RV function (see Supplementary data online, *Table S3*). When quantifying the blood flow velocity through the tricuspid valve, similar results as for the LV were noted, with controls having a higher 4D_RV_-E (53 ± 9 cm/s vs. 46 ± 10 cm/s, *P* < 0.01) but lower 4D_RV_-A (32 ± 9 cm/s vs. 45 ± 17 cm/s, *P* < 0.01) in comparison to CVD patients. Age-adjusted group differences were also observed for the RV diastolic ratios, 4D_RV_-E/A (1.8 ± 0.6 vs. 1.1 ± 0.6, *P* < 0.01), 4D_RV_-E/e’ (19 ± 6 vs. 29 ± 11, *P* < 0.01), 4D_RV_-E/e’_FT-SR_ (255 ± 122 ± vs. 271 ± 123, *P* = 0.02), and 4D_RV_-E/e’_FT-Vel_, (7.1 ± 3.9 vs. 7.0 ± 3.6, *P* = 0.02). No RV diastolic assessments were available from the clinical echocardiography reports. Thus, using cutoffs derived from the 80th (lower) and 97.5th (upper) percentile of healthy control values, a lower cutoff of 4D_RV_-E/e’>4.3 identified 61% of the second CVD subgroup as having possible RV-DD, with 51% exceeding the upper cutoff of 4D_RV_-E/e’>5.9.

### Inter-reader reliability

Good to excellent ICC were calculated for the diastolic parameters of both ventricles (see Supplementary data online, *Table S6*). Specifically, for LV 4D-E/e’, ICC was 0.94 (95%CI: 0.80–0.98, *P* < 0.01) and 4D-E/e’_RV_ ICC was 0.91 (95%CI: 0.71–0.97, *P* < 0.01).

## Discussion

By combining CMR 4D flow-derived transvalvular blood flow velocity (4D-E) along with tissue relaxation (e’) quantified by strain and valvular displacement, we derived the diastolic marker 4D-E/e’ for both ventricles. Our CMR-derived metrics demonstrated that CVD patients with chronic coronary syndromes and/or heart failure exhibited significantly impaired biventricular 4D-E/e’ values compared to healthy controls, even after adjusting for age. Importantly, over half of the CVD patients had not undergone any clinical assessment of diastolic function, despite receiving recent cardiovascular evaluations. Among this group, 71% were found to have abnormal LV diastolic function when stratified using a lower 4D-E/e’ cutoff >5.8, with 51% exceeding an upper threshold 4D-E/e’>7.3. Furthermore, CMR enabled concurrent assessment of RV diastolic function, which is often overlooked with clinical assessments.

### LV diastolic assessment using tissue or blood flow velocity separately

For our investigation, the e’ measurement derived from valvular displacement showed moderate agreement with echocardiographic tissue Doppler imaging, consistent with previous findings.^13^ Significant differences were observed in tissue relaxation parameters (e’, e’_FT-SR_ and e’_FT-Vel_) between the controls and CVD patients. These tissue-based metrics still alone demonstrated reasonable discriminatory performance for identifying DD. This is valuable as echocardiography algorithms often begin by evaluating e’ before progressing to transmitral flow measurements, and a similar pathway could be chosen with CMR.

With respect to transvalvular blood flow measurements, both 4D-E and 4D-E/A correlated well with echocardiography. There are differences in how flow is measured by each technique, which could account for minor discrepancies. Echocardiography conventionally assesses transmitral inflow at the leaflet tips with a static plane, whereas we assess transmitral flow directly at the annulus adjusting for annular angle at each cardiac phase. This approach has been reported to provide greater accuracy and reproducibility than measuring 4D-flow transmitral velocities at the leaflet tips using static methods.^24^ Recent 4D flow studies investigating diastolic function have employed kinetic energy, or mitral inflow, often showing good agreement with echocardiography.^16–18^ While E/A ratio remains valuable, it has limitations, especially in cases using prospective gating or in patients with arrhythmias, where the A-wave may be absent.^21^ As both CMR tissue velocity and CMR blood flow velocity measurements show individual potential for the first step of assessing diastolic function, a combined E/e’ measurement, integrating both features can typically achieve a more comprehensive and physiologically meaningful assessment.^13,12^

### Combined blood flow and tissue velocity markers

4D-E/e’, based on annular displacement, yielded the best diagnostic performance. This likely reflects its similarity to echocardiographic methods, which assess annular motion rather than global myocardial deformation. While the feature-tracking-based ratios (4D-E/e’_FT-SR_ and 4D-E/e’_FT-Vel_) had slightly lower AUCs, they still showed promising potential, and future work should investigate their relationship with invasive pressure measurements. The familiarity of E/e’ makes it a pragmatic clinical marker. Using a CMR 2D flow-derived E/e’, Fujikura et al. demonstrated good agreement in DD classification with echocardiography.^13^ Ramos et al. also utilized 2D-flow CMR and derived tissue velocities directly from their in-house designed sequence, reporting moderate correlations of E/e’ to echocardiography.^12^ Assessments of 4D-E/e’ in DD were recently introduced by Reiter et al.^15,20^ although using static planes. CMR-derived 4D-E/e’ is just emerging, and an advantage of our study is that we use adaptive valve tracking tools for transvalvular blood flow analysis accounting for annular angle and motion. Secondly, we demonstrate that both valvular velocities and strain measurements derived from standard cine images can be used, making the approach adaptable to different post-processing platforms and clinical settings. Thirdly, we also quantify the right ventricle (see Supplementary data online, *Table S1*).

### Assessment of RV diastolic function

**Figure 5:**
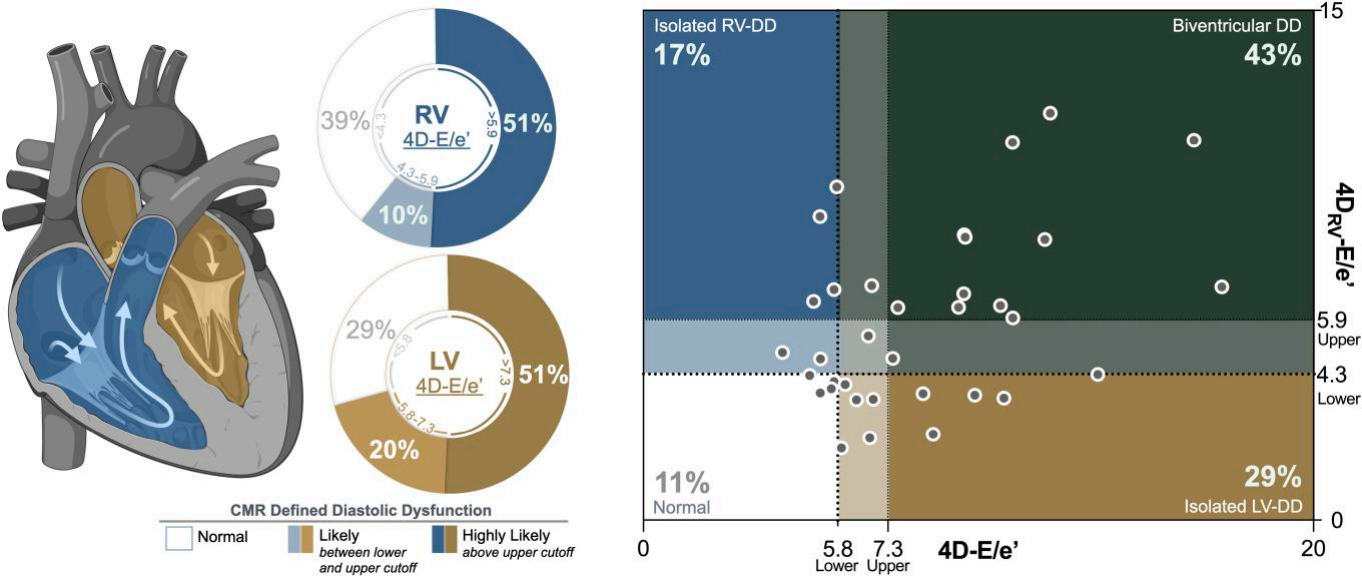
Not a mirror image, the importance of quantifying RV diastolic function. *Left*: Out of all patients who did not have an assessment of diastolic dysfunction (DD), 71% of patients had abnormal left ventricle (LV) diastolic function (bronze) defined by a lower cutoff 4D-E/e’>5.8, with 51% exceeding the higher cutoff of 4D-E/e’>7.3. Similarly, 61% had abnormal right ventricular diastolic function (blue) defined by an 4D_RV_-E/e’>4.3, with 51% yielding 4D_RV_-E/e’>5.9. *Right*: 4D-E/e’ values are plotted for the LV and RV, of which 43% had CMR defined biventricular diastolic dysfunction, 29% had isolated LV diastolic abnormalities where RV values were normal, 17% had isolated RV diastolic abnormalities, and 11% had normal diastolic findings in both ventricles (based on the lower cutoffs).

Evaluating RV diastolic function is inherently challenging due to the complex geometry and movement of the tricuspid valve, which 4D flow may help to overcome. Fenster et al.^25^ found correlations between RV-4D E and A wave vorticity and echocardiographic markers of RV-DD such as E/A or e’. In our study, we focused on transtricuspid blood flow velocity (4D_RV_-E and 4D_RV_-A), tricuspid annular velocity, and RV free wall strain. We could show a difference in RV diastolic function between controls and CVD patients, which may have subclinical RV dysfunction even in the absence of LV diastolic abnormalities (*Figure 5*). In 17 of 35 patients (subgroup 2), LV and RV-DD classifications based on the lower cutoff were discordant, emphasizing the importance of biventricular assessment and not just assuming RV diastolic function mirrors that of the left side. For example, some patients had normal LV diastolic function (4D-E/e’≤5.8), but abnormal RV diastolic function (4D_RV_-E/e'>4.3). This discrepancy may reflect physiological differences between the ventricles: RV diastolic function may be more susceptible to respiration, preload, and pulmonary resistance, while the LV diastolic parameters are more impacted by age, arterial hypertension, and ventricular hypertrophy.^7,26,27^ The gold standard for RV-DD remains invasive catheter-based assessment.^28^ Not one clinical report from our patients graded RV-DD, thus we are not able to assess the diagnostic accuracy of these cutoffs. As longitudinal deformation in the RV can detect early changes in the progression of DD in HFpEF (heart failure with preserved ejection fraction) patients,^29^ the individual use or combination of strain or annular excursion techniques with tricuspid 4D flow analysis provides a promising method to assess RV-DD.

### Clinical implications of CMR for highlighting unknown diastolic abnormalities

**Figure 6:**
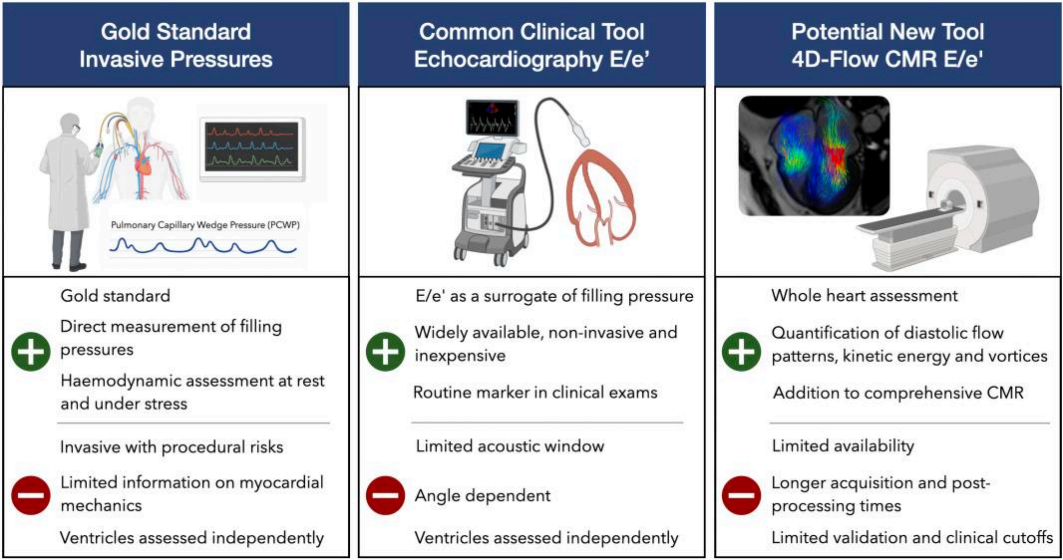
Comparison of diastolic function analysis techniques. Invasive pressure measurements represent the gold standard for the assessment of diastolic function, while echocardiography is the most widely used and readily available clinical tool. CMR offers distinct and complementary advantages and may serve as a first-line method for identifying diastolic dysfunction in patients already undergoing CMR for other clinical indications, with targeted follow-up using echocardiography or invasive assessment as appropriate. CMR, cardiovascular magnetic resonance.

The addition of 4D flow imaging allows for comprehensive biventricular diastolic assessment during a CMR exam. Although CMR-based diastolic evaluation is not yet a routine clinical tool, a key goal is to use CMR to highlight abnormal diastolic function in patients undergoing CMR for other indications, typically involving structural or myocardial tissue assessments. In this setting, CMR may serve as an initial screening tool to highlight potential diastolic abnormalities. If clinically indicated, patients could be subsequently referred for targeted echocardiographic evaluation or invasive catheter-based assessment until CMR-based diastolic metrics are more comprehensively validated (*Figure 6*). Therefore, for both ventricles, we provide two cutoffs: a lower threshold (4D-E/e’>5.8, 4D_RV_-E/e’>4.3) which has higher sensitivity and indicates that diastolic abnormalities may be present and warrants further evaluation; and an upper threshold (4D-E/e’>7.3, 4D_RV_-E/e’>5.9), which defines a higher likelihood of DD. Using these thresholds, two-thirds of the CVD patients without prior diastolic evaluation had abnormal LV-4D-E/e’ based on the lower cutoff, and still more than half exceeded the upper cutoff, with similar findings observed for the RV. A large study showed that among patients with coronary heart disease and no history of heart failure, 36% had asymptomatic LV-DD, which was associated with poorer outcomes.^5^ This highlights that detecting early diastolic abnormalities, even if it is not the primary indication, may enable timely risk stratification and treatment strategies.

### Considerations in the interpretation of CMR-derived diastolic markers

Our study focuses on E/e’, but as with all imaging markers of diastolic function, its utility is influenced by clinical context, and marker selection should be individualized as there are benefits but also confounders for various imaging markers. E/e’ may be uninformative in certain cases^30^; for instance, in the setting of moderate to severe mitral regurgitation, or restrictive cardiomyopathies where E/e’ is falsely low and not predictive of invasively measured PCWP.^31^ In HFpEF, E/e’ was shown to be unreliable during diastolic stress echocardiography, and it was suggested in this situation to rely on e’.^30^ Our study also highlights the utility of e’, which performed nearly as well as E/e’. Because it is derived from routine cine images it is widely available and doesn’t require advanced flow sequences or specialized software.

Alternative CMR-derived markers, including 4D-Flow based pulmonary metrics and CMR estimates of PCWP, have been validated against right-heart catheterization measures and may serve as alternative or complementary measures.^12,22,32^ CMR-PCWP is among the most extensively validated CMR-derived indices against invasively confirmed raised filling pressures; however its comparative accuracy varies by population and disease stage.^22,32,33^ Because CMR-PCWP is derived from ventricular mass and atrial volume, it primarily reflects structural remodelling and may be less sensitive in early-stage DD and HFpEF, where chamber dimensions may remain within reference ranges despite functional impairment.^30,33^ This likely explains the weaker association of CMR-PCWP with echocardiography-based DD classification and with 4D-E/e’ in our predominantly grade I DD and HFpEF cohort (see Supplementary data online, *Data S3*). Reliance on anatomical measures may also lead to overestimation of CMR-PCWP in conditions associated with physiological or pathological left atrial enlargement, such as athletic adaptation, atrial fibrillation, or valvular disease, where function-based markers, like 4D-E/e’, may be particularly informative.

The performance of individual CMR-derived markers is likely influenced by disease stage and remodelling patterns. Early DD is typically characterized by increased ventricular stiffness and reduced e’, with E/e’ rising as the disease progresses. In more advanced stages, elevated filling pressures and atrial remodelling become increasingly prominent, reflected by higher PCWP and E/e′ values.^10,34,35^ The optimal marker for different stages of diastolic dysfunction remains to be established and will require evaluation in larger, stage-stratified cohorts. We envisage that 4D-E/e′ and CMR-PCWP may ultimately be regarded as complementary CMR-based tools, with 4D-E/e′ offering greater sensitivity to early functional abnormalities and CMR-PCWP refining assessment in later, remodelling-driven disease. Having multiple CMR-derived options that can be applied individually or in combination may help ensure clinically relevant diastolic dysfunction is not overlooked.

### Limitations and future considerations

As echocardiographic measurements were obtained from clinical reports, not all patients underwent imaging concurrently with CMR, and haemodynamic conditions may have differed between exams. Furthermore, the echocardiographic measurements and a clinical grading of diastolic function were only available in a part of the patient cohort. Future studies will have to compare CMR 4D_RV_-E/e’ with invasive reference standards, establishing validity to elaborate a systematic approach to CMR based RV diastolic assessment as our cutoffs are based on reference intervals from healthy controls. This analysis statistically accounts for age differences among the controls; however, age and sex related reference values should be determined to avoid these biases. In addition, the proposed cutoffs were designed to maximize sensitivity and catch possible abnormalities; comparison with invasive measurements will be required to establish thresholds suitable for definitive diagnosis. Future assessments can investigate if whole heart algorithms can improve diagnosis of DD, potentially including newer techniques such as atrial strain, or 4D flow pressure analysis.^10,12,21^

## Conclusion

CMR-derived 4D-E/e’ ratios, using annular displacement or strain analysis, enable quantification of biventricular diastolic function by integrating blood flow and tissue velocity. This approach could fill a diagnostic gap in CMR, which has traditionally focused on systolic function and tissue characterization. Our results support further developing the clinical utility of CMR in identifying abnormal diastolic function of both the left and right ventricle, and further research is warranted to validate these measurements against reference standards and refine its diagnostic role.

## Supplementary Material

qyag039_Supplementary_Data

## Data Availability

The data underlying this article will be shared on reasonable request to the corresponding author.

## Lead author biography

Leonard Grob, MD **is a resident** in internal medicine with a research focus on cardiovascular imaging. He completed his medical training at the University of Bern, where his doctoral work investigated applications of 4D flow imaging in anaesthesia. He continued this work through a postdoctoral research fellowship at the Department of Anaesthesiology and Pain Medicine, Inselspital, Bern University Hospital, focusing on the integration of cardiology and radiology imaging techniques into anaesthesia research. He is currently expanding his research towards multidisciplinary cardiovascular imaging. His clinical and research interests include diastolic heart failure, clinical applications of 4D flow CMR, and myocardial strain imaging, with the goal of improving diagnostic accuracy and patient-centred imaging strategies in cardiovascular disease.
