# Supplementary material for: Evaluating biventricular diastolic function using cardiovascular magnetic resonance 4d-flow derived E/e’
Source: Eur Heart J Imaging Methods Pract. 2026 Mar 9;4(1):qyag039. doi: 10.1093/ehjimp/qyag039 (PMC12994140; doi:10.1093/ehjimp/qyag039)
Supplement: qyag039_Supplementary_Data [file qyag039_supplementary_data.zip › Supplemental_Grob_4D_08032026.pdf]

# SUPPLEMENTAL MATERIAL

# Evaluating Biventricular Diastolic Function Using Cardiovascular Magnetic Resonance 4D-Flow Derived E/e'

Table of Contents

SUPPLEMENTAL DATA 1: SEQUENCE PARAMETERS & IMAGE INCLUSION .......... 2

SUPPLEMENTAL DATA 2: METHODS FOR QUANTIFICATION OF TRANSVALVULAR BLOOD FLOW 3

SUPPLEMENTAL DATA 3: CMR DERIVED ESTIMATES OF PULMONARY CAPILLARY WEDGE PRESSURE (PCWP) .......... 4

SUPPLEMENTAL TABLE 1: USE OF CMR E/E' IN THE LITERATURE .......... 7

SUPPLEMENTAL TABLE 2: PATIENT CHARACTERISTICS .......... 9

SUPPLEMENTAL TABLE 3: STANDARD VOLUMETRIC MEASUREMENTS .......... 11

SUPPLEMENTAL TABLE 4: DIAGNOSTIC POTENTIAL OF TISSUE METRICS (E') .......... 12

SUPPLEMENTAL TABLE 5: ROC AND CUTOFF FOR E/E' IN AGE-ADJUSTED CONTROLS .......... 13

SUPPLEMENTAL TABLE 6: INTEROBSERVER INTRACLASS CORRELATION COEFFICIENTS .......... 14

**Supplemental Data 1: Sequence Parameters & Image Inclusion**

Imaging parameters for the prospective 4D flow sequence included a temporal resolution of 40.8ms, an acquired voxel size of 2.25x3.08x3.00 mm3 and 52-64 slices per slab with a k-t GRAPPA acceleration factor of 5. For the retrospective gated 4D flow CMR exam the phase number was predefined based on the RR-interval, yielding a typical reconstruction repetition time of 37-42ms, with a spatial resolution of 2.25x2.75x2.50mm3, 56-80 slices per slab and a compressed sensing acceleration factor of 7.6.

Left ventricular feature tracking strain analysis could be performed in all participants, while for right ventricle, two datasets couldn’t be assessed for strain due to tracking errors.

**Supplemental Data 2: Methods for Quantification of Transvalvular Blood Flow**

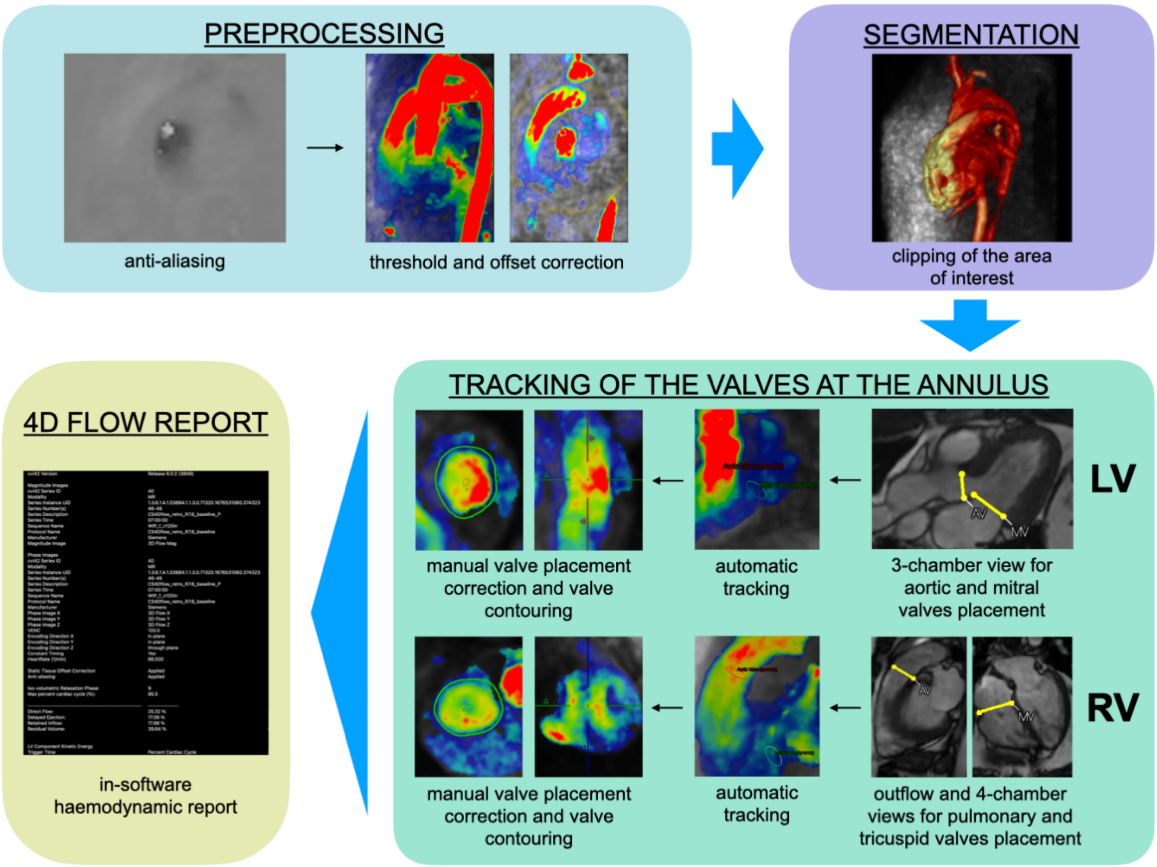


Detailed step-by-step process of 4D-Flow image analysis and acquisition of E and A velocity. LV = left ventricle. RV = right ventricle

## Supplemental Data 3: CMR Derived Estimates of Pulmonary Capillary Wedge Pressure (PCWP)

**Analysis Technique:**

A non-invasive estimate of PCWP, reflecting filling pressures, was calculated by implementing the following sex-adjusted formula introduced by Garg et al, 2024:

Sex-specific CMR PCWP = 5.7591 + (0.07505 x left atrial volume) + (0.05289 x left ventricular mass) – (1.9927 x sex) [female = 0; male = 1]

**Controls vs Patients with Cardiovascular Disease (CVD)**

CMR-PCWP could be calculated in 130 participants. Foreshortened planes led to exclusion of a few left atrial volume assessments. Controls were found to have a significantly lower sex-adjusted calculated CMR-PCWP than CVD-patients (17.0±2.2mmHg vs 18.4±4.2mmHg, p<0.01).

**Comparison of CMR-PCWP to Clinical Diagnosis of Diastolic Dysfunction (DD)**

First, logistic regression and a receiver operating curve analysis were performed (similar to the primary statistics) to assess if CMR-PCWP could discriminate the patients with DD. As shown in *panel A*, CMR-PCWP could not discriminate the cohort, consisting of primarily DD grade I. A secondary analysis *(panel B*) plotted the CMR-PCWP for controls and CVD patients categorized by their clinical diagnosis. It can be observed that patients with no DD or grade 1 have the same CMR-PCWP as controls. Only with higher grades of DD there is a visual increase, but still variation, potentially due to the confounding geometry of patients with Heart Failure and preserved Ejection Fraction (HFpEF).

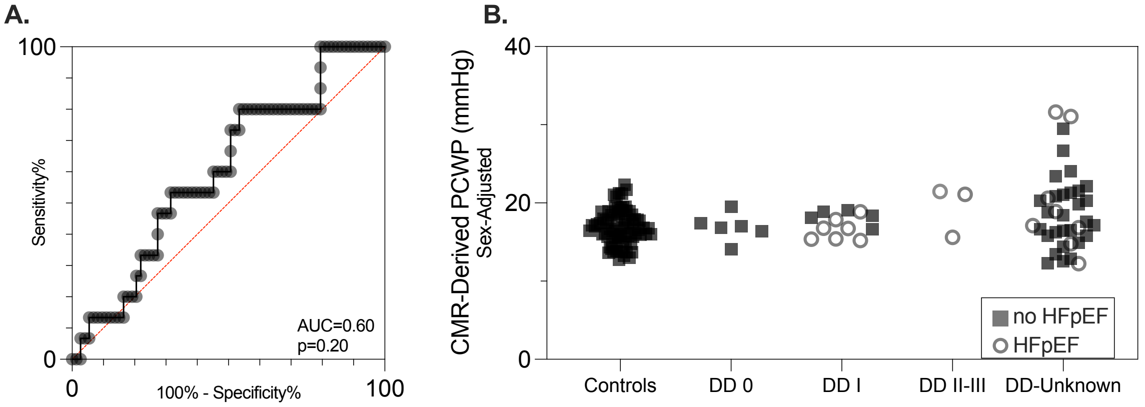


**Comparison of Left Ventricular 4D-E/e' to Sex-Adjusted CMR-PCWP**

Similar analysis was performed comparing the diagnostic ability of 4D-E/e' to detect participants with an elevated CMR-PCWP≥17.5mmHg. AUC analysis yielded a non-significant association between (*Panel C*, AUC=0.60,

p=0.054) 4D-E/e’ and patients with elevated CMR-PCWP (CMR-PCWP < or ≥17.5mmHg). A subsequent t-test demonstrated 4D-E/e’ was significantly lower (5.9±2.4) in participants with CMR-PCWP<17.5mmHg than in patients with elevated CMR-PCWP≥17.5mmHg (7.3±3.7, p=0.02, *Panel D*).

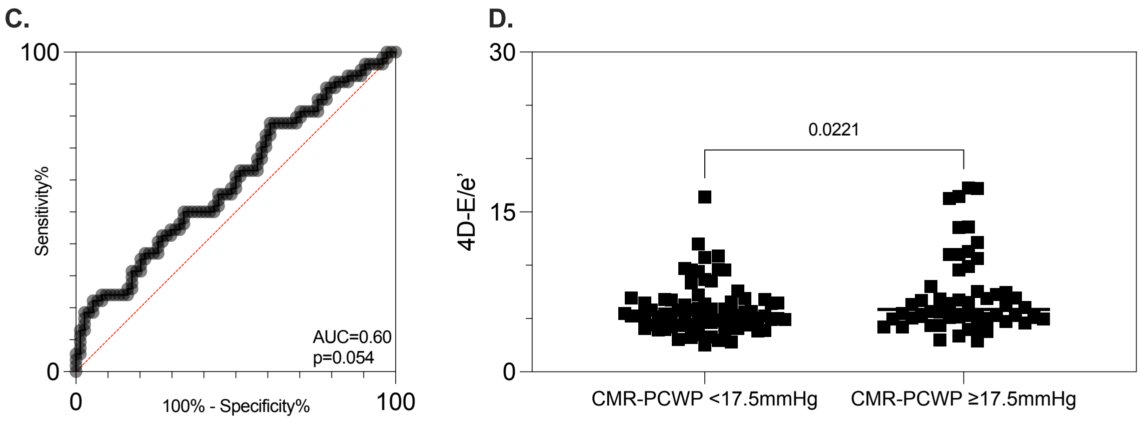


**Impact of Left Ventricle Size (End-Diastolic Volume) on Sex-Adjusted CMR-PCWP and 4D-E/e’**

Scatterplots and correlation analysis demonstrate that 4D-E/e’ has no association with left ventricular chamber size, assessed by end-diastolic volume (*panel E*), while there is a significant correlation with CMR-PCWP and end-diastolic volume (*panel F*). This supports the theory that while CMR-PCWP is an ideal non-invasive marker of filling pressures, it is confounded by the heart size and is thus not as ideal a marker in patients with so called “smaller hearts” such as those observed in HFpEF (Backhaus et al, 2024, Garg et al 2022, Baritussio et al 2022).

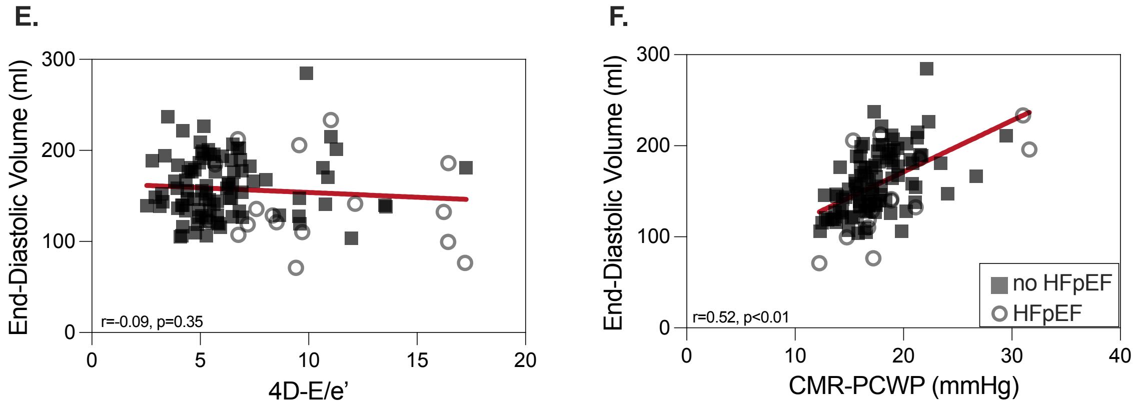


**Clinical Outlook**

Further studies, implementing both 4D-E/e’ and CMR-PCWP in a cohort with evenly spread diastolic dysfunction grades are warranted to fully reveal the relationship between these non-invasive markers and to correlate them to invasive measurements. As both markers have their strengths and weaknesses in certain scenarios, assessment of both markers may be optimal based on the supposed severity of diastolic dysfunction, since they can easily be acquired within the same CMR scan.

**References**

- *Garg P, Gosling R, Swoboda P, Jones R, Rothman A, Wild JM, et al. Cardiac magnetic resonance identifies raised left ventricular filling pressure: prognostic implications. Eur Heart J. 2022 May 4;43(26):2511–22.*
- *Garg P, Grafton-Clarke C, Matthews G, et al. Sex-specific cardiac magnetic resonance pulmonary capillary wedge pressure. Eur Heart J Open 2024;4:oeae038.*
- *Backhaus, S.J., Schulz, A., Lange, T. et al. Real-time cardiovascular magnetic resonance imaging for non-invasive characterisation of heart failure with preserved ejection fraction: final outcomes of the HFpEF stress trial. Clin Res Cardiol. 2024, 113, 496–508.*
- *Anna Baritussio, Vivek Muthurangu, Cardiovascular magnetic resonance for the assessment of left ventricular filling pressure in heart failure,* European Heart Journal*, Volume 43, Issue 26, 7 July 2022, Pages 2523–2525.*

## Supplemental Table 1: Use of CMR E/e' in the literature

| **Reference** | **E (transvalvular blood flow)** | **e' (tissue)** | **Comments and comparison to the applied technique** |
|---|---|---|---|
| **4D Flow** | | | |
| Grob et al.: Techniques proposed in current manuscript | ***Sequence:* 4D Flow**<br><br>*Measurement:*<br>Blood flow velocity at annulus using valve-motion correction | ***Sequence:* 2D cine**<br><br>*Measurement:*<br>A) tissue velocity of mitral valve (lateral and septal) and tricuspid valve in long-axis view<br><br>B) longitudinal strain rate (left ventricle, and right ventricular free wall) assessed by feature tracking<br><br>C) longitudinal strain rate velocity (left ventricle, and right ventricular free wall) assessed by feature tracking | |
| Reiter et al. 2024, 2025<br>1)DOI: 10.1016/j.ejrad.2023.111247<br><br>2)DOI: 10.1007/s00330-025-11703-0 | ***Sequence:* 4D Flow**<br><br>*Measurement:*<br>A) Static plane for blood flow velocity at leaflet tips<br>B) Static plane for quantifying highest velocity between annulus and leaflet tips | ***Sequence:* 4D Flow**<br><br>*Measurement:*<br>Tissue velocity (septal and lateral in long-axis view) | -Flow was assessed using a static plane not accounting for through-plan motion<br><br>-Tissue metrics were calculated directly from 4D dataset |
| Varga-Szemes, 2022<br>DOI: doi.org/10.1002/jmri.28484 | ***Sequence:* 4D Flow**<br><br>*Measurement:*<br>Blood flow velocity at annulus using valve-motion correction using built-in algorithms | ***Sequence:* 4D Flow**<br><br>*Measurement:* undefined | -Show reproducibility of E/e' between two 4D flow sequences in healthy controls.<br><br>-Tissue metrics were calculated directly from 4D dataset |
| **2D Flow** | | | |
| Fujikura et al. 2024<br>DOI: 10.1038/s41598-024-61992-6 | ***Sequence:* 2D phase-contrast** | ***Sequence:* 2D cine** | -Flow based on 2D |

| | | | |
|---|---|---|---|
| | *Measurement:*<br>Maximum blood flow velocity measured in mitral valve short-axis plane | *Measurement:*<br>Tissue velocity of mitral valve (lateral and septal) and tricuspid valve in long-axis view | -No assessment of right heart |
| Fyrdahl et al 2019,<br>DOI: 10.1002/mrm.28018<br><br>Ramos et al. 2025<br>DOI: 10.1016/j.jcmg.2020.06.027 | ***Sequence:* 2D high–temporal resolution sector-wise golden-angle phase-contrast sequence**<br><br>*Measurement:*<br>Maximum blood flow velocity measured in mitral valve short-axis plane | ***Sequence:* 2D high–temporal resolution sector-wise golden-angle phase-contrast sequence**<br><br>*Measurement:*<br>Tissue velocity of septal and lateral wall in short-axis view | -Flow based on 2D<br>-No assessment of right heart<br><br>*Ramos et al used additional 4D flow sequence for assessment of the pulmonary artery |
| Lamy et al 2024<br>DOI: 10.14814/phy2.70078 | ***Sequence:* 2D phase-contrast**<br><br>*Measurement:*<br>Maximum blood flow velocity through mitral valve in long-axis view | ***Sequence:* 2D cine**<br><br>*Measurement:*<br>Tissue velocity of mitral valve (lateral and septal) and tricuspid valve in long-axis view | -Flow based on 2D<br>-No assessment of right heart |
| Ouyang et al. 2024<br>DOI: 10.1007/s00330-023-10538-x | ***Sequence:* 2D phase-contrast**<br><br>*Measurement:*<br>Maximum blood flow velocity through tricuspid valve perpendicular to right ventricular inflow | ***Sequence:* 2D cine**<br><br>*Measurement:*<br>Right ventricular free wall velocity assessed by feature tracking | -Flow based on 2D<br>-Assessed the right heart, but not the left |

The table depicts literature which have published a CMR derived E/e’ ratio using 2D or 4D approaches. The sequences and key measurements used to derive the E/e’ ratios are provided, along with a short description of how the techniques differ.

While multiple publications have assessed transvalvular flow (E) or tissue velocities (e’) independently, few have assessed the combined ratio (E/e’), especially when it comes to using 4D flow. As the table depicts, different techniques have been applied, with none using the same approach used in this submission, nor assessing the right heart with 4D flow.

**Supplemental Table 2: Patient Characteristics**

| | **Controls (n=75)** | **Patients (n=57)** |
|---|---|---|
| Age (years) | 32±12 | 65±11 |
| Males | 42 (56%) | 47 (82%) |
| Body Mass Index (kg/m$^2$) | 23.6±3.0 | 29.4±5.7 |
| **Comorbidities** | | |
| Coronary Artery Disease | | 33 (58%) |
| Diastolic Dysfunction* | | |
| • All Grades | | 21 (37%) |
| • Grade 0 | | 6 (11%) |
| • Grade 1 | | 12 (21%) |
| • Grade 2 | | 0 (0%) |
| • Grade 3 | | 3 (5%) |
| Diabetes Mellitus | | 10 (18%) |
| Dyslipidemia | | 29 (51%) |
| Hypertension | | 22 (39%) |
| Arrythmia | | 17 (30%) |
| Valvular Disease | | 2 (4%) |
| **Medication** | | |
| Anticoagulants | | 18 (32%) |
| Anti-Platelets | | 33 (58%) |
| Beta Blockers | | 44 (77%) |
| ACE Inhibitors | | 25 (44%) |
| Angiotensin II Receptor Blockers | | 20 (35%) |
| Calcium Channel Blockers | | 21 (37%) |
| Diuretics | | 21(37%) |
| Statins | | 39 (68%) |
| Oral Antidiabetics | | 5 (9%) |
| Insulin | | 5 (9%) |

Mean±SD or frequency (percentage of group) are reported. ACE: angiotensin-converting enzyme. *Diastolic dysfunction graded/reconfirmed in the previous 90 days.

**Supplemental Table 3: Standard Volumetric Measurements**

| | Controls (n=75) | CVD Patients (n=57) | p |
|---|---|---|---|
| Heart Rate (bpm) | 64±12 | 68±17 | 0.17 |
| **Left Ventricle** | | | |
| End Diastolic Volume Index (ml/m$^2$) | 85±13 | 74±17 | <0.01 |
| Stroke Volume Index (ml/m$^2$) | 50±8 | 43±11 | <0.01 |
| Ejection Fraction (%) | 59±5 | 55±11 | 0.03 |
| Ejection Fraction (<45%) | 0 (0%) | 8 (11%) | <0.01 |
| Cardiac Index (l/min/m$^2$) | 3.2±0.8 | 2.7±0.8 | <0.01 |
| Aortic Peak Velocity (cm/s) | 120±17 | 134±31 | 0.01 |
| **Right Ventricle** | | | |
| End Diastolic Volume Index (ml/m$^2$) | 97±15 | 84±22 | 0.09 |
| Ejection Fraction <40% or Fractional Area Change <30% | 1 (1%) | 7 (9%) | <0.01 |
| Main Pulmonary Artery Peak Velocity (cm/s) | 85±13 | 88±22 | 0.51 |

Mean±SD of healthy controls and patients. All comparisons are adjusted for age.

## Supplemental Table 4: Diagnostic Potential of Tissue Metrics (e')

| | **Left Ventricle** |
|---|---|
| **e' (cm/s)** | 1.0 (0.0), p<0.01 |
| **$e'_{FT\text{-}SR}$ (/s)** | 0.91 (0.05), p<0.01 |
| **$e'_{FT\text{-}vel}$ (cm/s)** | 0.86 (0.05), p<0.01 |

Area under the curve (±standard error) along with the p-value is shown for the ability of the CMR measurements of tissue velocity from a standard four-chamber cine to discriminate patients with clinically diagnosed diastolic dysfunction (≥grade I) in comparison to control values. e' = average early diastolic mitral/tricuspid annular tissue velocity. $e'_{FT\text{-}SR}$ = early diastolic strain rate, $e'_{FT\text{-}vel}$ = early diastolic strain rate velocity

## Supplemental Table 5: ROC and Cutoff for E/e' in age-adjusted controls

| | AUC | Cutoff | Specificity (%) /Sensitivity (%) | CMR defined DD (n, %) |
|---|---|---|---|---|
| LV 4D-E/e' | 0.87 (0.09), p<0.01 | **Lower Cutoff** | | |
| | | >6.5 | 80% / 82% | 20/34 (59%) |
| | | **Upper Cutoff** | | |
| | | >6.9 | 91% / 70% | 16/34 (47%) |
| RV 4D-E/e' | - | **Lower Cutoff** | | |
| | | >5.7 | - | 18/36 (51%) |
| | | **Upper Cutoff** | | |
| | | >6.4 | - | 14/36 (39%) |

A secondary analysis of cut-offs was performed only using a smaller subset of healthy controls aged 50 years or older (n=11) as the reference.

Area under the curve (±standard error) along with the p-value is shown for the ability of E/e' to discriminate patients with clinically diagnosed diastolic dysfunction (≥grade I) in comparison to older control values. Lower cutoffs are generated based on 80% sensitivity for the left heart, or 80$^{th}$ percentile of healthy control values for the right heart. Upper cutoffs are based on the 97.5$^{th}$ percentile of healthy controls aged 50 or above (n = 11) for both ventricles. As there was no reference standard for RV measures, no area under the curve analysis is provided.

It can be observed that in comparison to the primary cutoffs in Table 2, the cutoffs and AUC are similar, with the lower cutoffs for both ventricles being slightly higher than observed in Table 2.

## Supplemental Table 6: Interobserver Intraclass Correlation Coefficients

| | Left Heart | | Right Heart | |
|---|---|---|---|---|
| | **ICC (95%CI)** | **p** | **ICC (95%CI)** | **p** |
| **Transvalvular Blood Flow Velocities** | | | | |
| 4D-E (cm/s) | 0.95 (0.86-0.99) | <0.01 | 0.77 (0.40-0.93) | <0.01 |
| 4D-A (cm/s) | 0.97 (0.91-0.99) | <0.01 | 0.99 (0.97-0.99) | <0.01 |
| 4D-E/A | 0.90 (0.68-0.97) | <0.01 | 0.96 (0.88-0.99) | <0.01 |
| **Tissue Velocities** | | | | |
| e' (cm/s) | 0.85 (0.55-0.95) | <0.01 | 0.86 (0.59-0.96) | <0.01 |
| $e'_{FT-SR}$ (/s) | 0.99 (0.96-0.99) | <0.01 | 0.87 (0.61-0.96) | <0.01 |
| $e'_{FT-vel}$ (cm/s) | 0.97 (0.91-0.99) | <0.01 | 0.86 (0.59-0.96) | <0.01 |
| **Transvalvular Blood Flow / Tissue Velocity Ratios** | | | | |
| 4D-E/e' | 0.94 (0.80-0.98) | <0.01 | 0.91 (0.71-0.97) | <0.01 |
| 4D-E/$e'_{FT-SR}$ (cm) | 0.99 (0.95-0.99) | <0.01 | 0.87 (0.62-0.96) | <0.01 |
| 4D-E/$e'_{FT-vel}$ | 0.99 (0.97-0.99) | <0.01 | 0.83 (0.52-0.95) | <0.01 |

Inter-rater reliability between two readers was evaluated using a two-way intraclass correlation coefficient (ICC) for absolute agreement (n=12). e' = average early diastolic mitral/tricuspid annular tissue velocity. $e'_{FT-SR}$ = early diastolic strain rate, $e'_{FT-vel}$ = early diastolic strain rate velocity
